# Electrochemical Fabrication of rGO-embedded Ag-TiO_2_ Nanoring/Nanotube Arrays for Plasmonic Solar Water Splitting

**DOI:** 10.1007/s40820-019-0329-2

**Published:** 2019-11-07

**Authors:** Lixia Sang, Lei Lei, Clemens Burda

**Affiliations:** 1 Key Laboratory of Enhanced Heat Transfer and Energy Conservation, Ministry of Education and Key Laboratory of Heat Transfer and Energy Conversion, Beijing Municipality, College of Environmental and Energy Engineering, Beijing University of Technology, Beijing, 100124 People’s Republic of China; 2 Department of Chemistry, Center for Chemical Dynamics and Nanomaterials Research, Case Western Reserve University, 10900 Euclid Avenue, Cleveland, OH 44106 USA

**Keywords:** TiO_2_ nanoring/nanotube hierarchical structure, Reduced graphene oxide, Spectral responses, Plasmonic Ag nanoparticles, Water splitting

## Abstract

Effective utilization of hot electrons generated from the decay of surface plasmon resonance in metal nanoparticles is conductive to improve solar water splitting efficiency. Herein, Ag nanoparticles and reduced graphene oxide (rGO) co-decorated hierarchical TiO_2_ nanoring/nanotube arrays (TiO_2_ R/T) were facilely fabricated by using two-step electrochemical anodization, electrodeposition, and photoreduction methods. Comparative studies were conducted to elucidate the effects of rGO and Ag on the morphology, photoresponse, charge transfer, and photoelectric properties of TiO_2_. Firstly, scanning electron microscope images confirm that the Ag nanoparticles adhered on TiO_2_ R/T and TiO_2_ R/T-rGO have similar diameter of 20 nm except for TiO_2_ R-rGO/T. Then, the UV–Vis DRS and scatter spectra reveal that the optical property of the Ag-TiO_2_ R/T-rGO ternary composite is enhanced, ascribing to the visible light absorption of plasmonic Ag nanoparticles and the weakening effect of rGO on light scattering. Meanwhile, intensity-modulated photocurrent spectroscopy and photoluminescence spectra demonstrate that rGO can promote the hot electrons transfer from Ag nanoparticles to Ti substrate, reducing the photogenerated electron–hole recombination. Finally, Ag-TiO_2_ R/T-rGO photoanode exhibits high photocurrent density (0.98 mA cm^−2^) and photovoltage (0.90 V), and the stable H_2_ evolution rate of 413 μL h^−1^ cm^−2^ within 1.5 h under AM 1.5 which exceeds by 1.30 times than that of pristine TiO_2_ R/T. In line with the above results, this work provides a reliable route synergizing rGO with plasmonic metal nanoparticles for photocatalysis, in which, rGO presents a broad absorption spectrum and effective photogenerated electrons transfer.

## Highlights

- Reduced graphene oxide (rGO) in electrode can weaken the light scattering of plasmonic Ag nanoparticles and promote the hot electrons transfer from Ag nanoparticles to Ti substrate.
- A route synergizing rGO with plasmonic Ag on TiO_2_ for plasmonic solar water splitting was provided.

## Graphical abstract

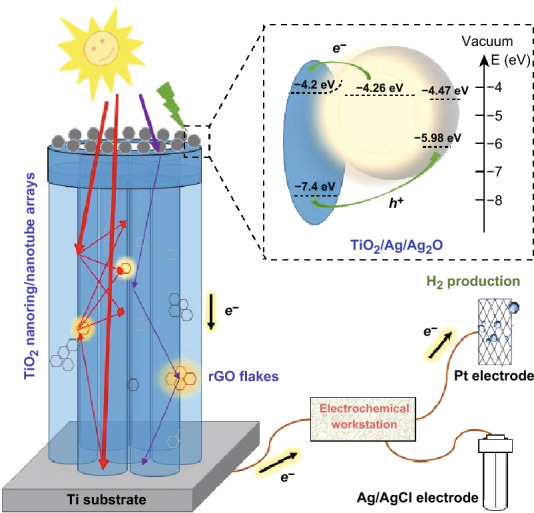

## Introduction

Nowadays, more and more researchers have paid attention to the preparation and application of nanomaterials. Strong photoresponse in the visible light region allows plasmonic metal nanoparticles to serve as tunable light antennas of wide-bandgap semiconductors, such as TiO_2_, for overcoming the limitations of rapid recombination of photogenerated electron–hole pairs and inability to absorb visible light [1]. Among the plasmonic metals, Ag nanocrystals have attracted considerable attention for their remarkable plasmon resonance enhancement. Lian et al. [2] designed a plasmonic Ag/TiO_2_ photocatalytic composite by selecting Ag quantum dots to act as the photosensitizer for driving the visible light-driven photoelectrocatalytic hydrogen evolution. Chen et al. [3] deposited Ag nanoparticles on the flame reduced TiO_2_, which exhibits significant application prospects for the enhanced solar conversion efficiency. Mohammadi et al. loaded Ag nanoparticles on TiO_2_ nanotubes by sequential chemical bath deposition, and the photoelectrochemical (PEC) activity is considerably higher than the bare TiO_2_ (about 3 times), owing to the light absorption improvement of plasmonic effects in addition to better separation and transport of electron–hole pairs [4]. In addition, our research group has also probed plasmonic Ag nanoparticles on TiO_2_ nanotube arrays electrode for efficient solar water splitting [5]. Once Ag nanoparticles (Ag NPs) get in touch with TiO_2_, a Schottky barrier is formed and the height plays an important role in electron transfer [6]. Under the ultraviolet irradiation, the electrons belonging to the valence band of TiO_2_ migrate to its conduction band and then are transferred to Ag NPs with the help of the Schottky barrier. Under the visible light irradiation, Ag NPs exhibit surface plasmon resonance (SPR) effect, and a number of hot electrons are generated via Landau damping [7]. The high-energy hot electrons in Ag NPs can be transferred across the Schottky barrier to TiO_2_, followed by photocatalytic reaction [8, 9]. However, the short lifetime of hot electrons (< 160 fs) has been the main limiting factor of its effective utilization [10]. To make full use of plasmon-induced hot electrons, it is necessary to optimize the structure and composition of Ag/TiO_2_ [11].

Graphene, an extensively used two-dimensional nonmetallic conductive material, is considered as an ideal electron transfer medium in photocatalysis for its various advantages, such as good interfacial contact with adsorbents, excellent mobility of charge carriers, and large surface area [12]. As a derivative of graphene, reduced graphene oxide (rGO) has the similar structure and properties [13, 14]. Lang et al. [15] made rGO nanosheets as conductive “bridge” to help transferring the hot electrons from Ag of smaller work function to TiO_2_ of larger work function. Gao et al. [16] fabricated the composite photocatalyst with Ag and TiO_2_ nanoparticles wrapped by rGO sheets, in which, rGO was used as an electron acceptor. In most designs, both Ag and TiO_2_ are co-deposited on the surface of rGO nanosheets to form Ag/rGO/TiO_2_ ternary catalysts. The two-dimensional rGO nanosheets act as a good electron transporting bridge owing to its high electron mobility and *π*-electron conjugation [17, 18]. Besides, rGO can broaden the absorption spectrum of TiO_2_ [19]. For example, a well-organized rGO and Ag wrapped TiO_2_ nanohybrid was successfully achieved by Leong and his colleagues, and the wrapped rGO nanosheets promoted visible light shift toward red spectrum [20].

The Ag/rGO/TiO_2_ ternary nanocomposite is usually used in heterogeneous systems. For instance, Ong et al. developed hybrid organic PVDF-inorganic Ag-rGO-TiO_2_ nanocomposites for multifunctional volatile organic compound sensing and photocatalytic reaction [21]. Nasrollahzadeh prepared Ag/rGO/TiO_2_ nanocomposite for the reduction of 4-nitrophenol, Congo red and methylene blue in aqueous media [22]. Pant et al. synthesized a multifunctional Ag-TiO_2_/rGO powder for wastewater treatment [23]. Recently, Tian et al. [24] synthesized mesoporous TiO_2_/rGO/Ag by an electrostatic self-assembly approach and a photo-assisted reduction process for the degradation of methylene blue. As much as we know, there is little research on utilizing the Ag/rGO/TiO_2_ ternary catalysts in PEC system. In addition, there are so many methods to fabricate various conformations of Ag/rGO/TiO_2_ ternary composite, such as hydrothermal synthesis for 3D urchin-like Ag/TiO_2_/rGO composites [25], electrodeposit and chemical reduction methods for Ag/rGO co-decorated TiO_2_ nanotube arrays [26, 27], electrospinning technique for TiO_2_ nanofibers loaded on rGO/Ag platform [28], three-step method for TiO_2_-Ag-rGO vertical heterostructure [29], and so on, but the effect of rGO on the other two composites has been rarely discussed in these researches.

In the present work, TiO_2_ nanoring/nanotube arrays (TiO_2_ R/T) via two-step anodization were chosen as substrate electrode, which shows unique oscillating absorption in the visible region and excellent photoelectric properties in our previous work [30]. Herein, we focus on fabricating the TiO_2_ R/T electrodes with Ag NPs and rGO fragments to further improve the H_2_ production rate in PEC system. Different from other studies, rGO was added into the electrolyte of the first or second electrochemical anodization. Thus, the effects of rGO on morphology, photoresponse, charge transfer, and photoelectric properties of TiO_2_ and Ag in the nanocomposites were systematically investigated. Accordingly, the possible charge transfer routes and light absorption of as-prepared samples were discussed.

## Methods

### Materials

Ti foils (0.25 mm thick, 99.5% purity) were supplied by Alfa-Aesar Company. Aluminum oxide (Al_2_O_3_) was purchased from Gaona Powder Company (Shanghai, China). Graphite oxide powders were purchased from Xianfeng nanomaterials Company (Nanjing, China). Silver nitrate (AgNO_3_), sodium dodecyl sulfate (SDS), polyvinylpyrrolidone (PVP), and sodium borohydride (NaBH_4_) were purchased from Fuchen Chemical Reagent Company (Tianjin, China). Ethylene glycol (EG), sodium nitrate (NaNO_3_), ammonium fluoride (NH_4_F), sodium sulfate (Na_2_SO_4_), triethanolamine (TEOA), ethanol (C_2_H_6_O) and acetone (CH_3_COCH_3_), isopropyl alcohol (C_3_H_8_O) were purchased from Beijing Chemical Works (Beijing, China). All reagents were of analytical grade and used without further purification.

### Preparation of TiO_2_ R/T Modified with rGO in Nanoring or Nanotube

TiO_2_ R/T was prepared by using two-step anodization method in ethylene glycol solution with NH_4_F (0.25 wt%) and water (2 vol%) [30]. The first and second anodic oxidation steps correspond to the formation of nanorings and nanotubes, respectively. The graphene oxide (GO) solution of 0.75 g L^−1^ was prepared by adding given amount of GO powder to deionized water and sonicated for 5 h. A certain amount of SDS was added to enhance exfoliation and separate graphite oxide sheets from each other. Subsequently, 0.6970 g NH_4_F and 5 mL above GO-dispersed solution were dissolved in 250 mL ethylene glycol. The mixed solution was stirred uniformly. In the first anodization, Ti foil was anodized at 60 V for 60 min using ethylene glycol solution with NH_4_F and H_2_O as electrolyte, and the as-grown nanotube layer was ultrasonically removed in deionized water. After that, the pretreated Ti foil was used as anode again for the second anodization in the ethylene glycol solution with NH_4_F and GO-dispersed solution. Finally, the sample was reduced in 1 mol L^−1^ NaBH_4_ aqueous solution for 1 h with 300 W Xe-lamp irradiation and subsequently dried at 130 °C for 2 h in air. In the progress, the oxygen-containing functional groups on the surface of GO were removed and the *sp*^2^ hybrid orbits of carbon were re-created [31, 32]. The prepared sample is named as TiO_2_ R/T-rGO. The other sample, prepared in NH_4_F with rGO for the first anodization and NH_4_F with H_2_O for the second anodization, is named as TiO_2_ R-rGO/T.

### Deposition of Ag Nanoparticles

A two-electrode setup was used for pulse electrodeposition of Ag NPs by using TiO_2_ R/T, TiO_2_ R/T-rGO, and TiO_2_ R-rGO/T samples as the working electrodes, and Pt sheet as the counter electrode. The current pulsing approach was utilized via choosing a cathodic pulse (− 25 mA, 0.1 s) and a relation time (0.3 s) in a mixed solution containing 1 mM AgNO_3_, 10 mM NaNO_3_, 0.2 g L^−1^ PVP, and 0.8 vol% isopropyl alcohol with magnetic string at 200 rpm. The as-obtained products are labeled as Ag-TiO_2_ R/T, Ag-TiO_2_ R/T-rGO, and Ag-TiO_2_ R-rGO/T. The preparation conditions and corresponding samples are summarized and listed in Table 1.

**Table 1:** Preparation conditions of the TiO_2_-based samples

Samples	The electrolyte using in the first anodization	The electrolyte using in the second anodization	Deposition of Ag NPs
TiO_2_ R/T	NH_4_F + H_2_O + (CH_2_OH)_2_	NH_4_F + H_2_O + (CH_2_OH)_2_	No
Ag-TiO_2_ R/T	NH_4_F + H_2_O + (CH_2_OH)_2_	NH_4_F + H_2_O + (CH_2_OH)_2_	Yes
TiO_2_ R/T-rGO	NH_4_F + H_2_O + (CH_2_OH)_2_	NH_4_F + GO solution + (CH_2_OH)_2_	No
Ag-TiO_2_ R/T-rGO	NH_4_F + H_2_O + (CH_2_OH)_2_	NH_4_F + GO solution + (CH_2_OH)_2_	Yes
TiO_2_ R-rGO/T	NH_4_F + GO solution + (CH_2_OH)_2_	NH_4_F + H_2_O + (CH_2_OH)_2_	No
Ag-TiO_2_ R-rGO/T	NH_4_F + GO solution + (CH_2_OH)_2_	NH_4_F + H_2_O + (CH_2_OH)_2_	Yes

### Characterizations

The morphologies of TiO_2_ R/T hierarchical structures were characterized with a field-emission scanning electron microscope (FESEM; Hitachi, Japan, S4800). Transmission electron microscopies (TEM) were obtained with a JEM-2100 transmission electron microscope (Japan Electron Optics Laboratory Co., Ltd., JEOL) at an accelerating voltage of 200 kV. The Raman spectra were acquired using a confocal micro-Raman system (BRUKER, RFS100) with an excitation wavelength of 532 nm. The atomic force microscopic (AFM) measurement was performed with a custom-designed scanning probe microscope (NTEGRA Spectra, NTMDT), using a Co/Cr-coated silicon cantilever. The crystal phases of the samples were determined using an X-ray diffractometer (XRD; D8 Advance Bruker/AXS) with Co (30 mA) radiation. The diffraction data were recorded for 2*θ* between 5° and 80°. X-Ray photoelectron spectroscopy (XPS) (Escalab 250Xi, Thermo Fisher Co., USA) measurements were processed using an Al-Ka monochromatic X-ray source (1486.6 eV). All binding energies were referenced to the C 1 s peak at 284.8 eV of surface adventitious carbon. The optical absorption spectra were obtained by employing a Fiber Spectral Instrument (AVANTES, Netherlands) and compressed BaSO_4_ powder as reflectance standard. Photoluminescence (PL) studies were done using a Gilden Photonics photoluminescence spectrophotometer under 260 nm excitation. Scattering spectra were obtained under 410–590 nm excitation.

### Photoelectrochemical Measurements

The PEC performances were measured in a three-electrode system with TiO_2_-based composites, Ag/AgCl electrode, and Pt mesh as the working, reference, and counter electrodes, respectively. The supporting electrolyte was carried out in aqueous solution containing 15 vol% TEOA and 0.5 M Na_2_SO_4_. The samples were illuminated by a solar simulator (91160, Newport) which equipped with an AM 1.5 filter. The intensity was calibrated to 100 mW cm^−2^. The visible light activity was evaluated under chopped light irradiation with light wavelength ≥ 400 nm by using a filter (FSQ-GG400, Newport). The linear sweep voltammetry (LSV), photocurrent density–time (*I*-*t*), open-circuit photovoltage (*U*-*t*), and intensity-modulated photocurrent spectroscopy (IMPS) results were collected by an electrochemical workstation (AUTOLAB PGSTAT302 N). The wavelength of the irradiation light for IMPS measurements was set as 410 nm. Detection of hydrogen was performed online by using an Agilent 7890B gas-chromatograph (5A molecular sieve, N_2_ carrier gas).

### Computational Model and Methods

The general gradient approximate (GGA) with the PBE was adopted to estimate the optical properties of graphene using CASTEP code [33]. An energy cutoff of 750 eV has been used for expanding the Kohn–Sham wave functions. The SCF tolerance was set as 2.0 × 10^−6^ eV/atom. The k-points grid sampling of Monkhorst–Pack scheme was set as 1 × 1 × 1 in the irreducible Brillouin zone. Moreover, the fast Fourier transform grid was set as 48 × 48 × 48. The dipole corrections were utilized for the model, which are essential in eliminating the nonphysical electrostatic interaction between periodic images.

## Results and Discussion

### The Effect of rGO on the Compositions and Morphologies of Ag/TiO_2_

**Fig. 1:**
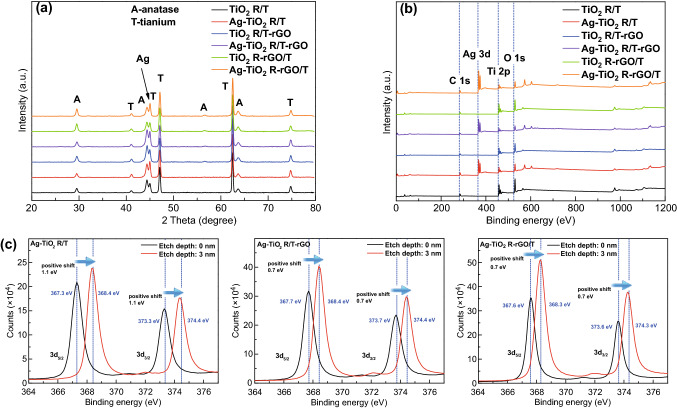
**a** XRD patterns, **b** XPS full spectrum scanning, and **c** high-resolution XPS spectra of the Ag 3d peaks of TiO_2_-based composites

The compositions of bare and modified TiO_2_ R/T were obtained by XRD patterns, as shown in Fig. 1a. For all samples, the diffraction peaks could be indexed to the tetragonal TiO_2_ anatase phase (JCPDS No. 21-1272) and the hexagonal Ti metal phase (JCPDS No. 44-1294). rGO and Ag have little effect on the crystal phase of TiO_2_ R/T. However, no rGO diffraction peaks could be identified due to its high dispersity and low loading, which may be below the detection limit of XRD [27]. The diffraction peaks ascribed to Ag NPs were also not clearly distinguished because the peak corresponding to Ag at 44.6° could be covered up by the peak of Ti at 44.9° [34]. Therefore, the XPS technique was employed to analyze the specific surface composition and elemental binding energy, as displayed in Fig. 1b. It is suggested that the composites contain C, Ag, Ti, and O, and the chemical binding energies located at 284.8, 368.7, 459.5, and 529.6 eV are assigned to the characteristic peaks of C 1s, Ag 3d, Ti 2p, and O 1s, respectively. Moreover, Fig. 1c exhibits the high-resolution spectrum of Ag 3d from the Ag-modified samples. The Ag 3d_5/2_ and Ag 3d_3/2_ core levels of Ag-TiO_2_ R/T, Ag-TiO_2_ R/T-rGO, and Ag-TiO_2_ R-rGO/T were fitted with two peaks at the binding energy of about 367 and 373 eV, respectively, corresponding to the Ag^+^ ions produced by the electron transfer between metallic Ag and TiO_2_/rGO [16, 29]. It should be noted that the characteristic peaks of the Ag 3d from Ag-TiO_2_ R/T-rGO and Ag-TiO_2_ R-rGO/T have positively shifted by 0.4 and 0.3 eV relative to Ag-TiO_2_ R/T, respectively, confirming that the electrons indeed transfer from Ag to rGO. Coincidentally, Shi et al. reported that the two peaks of Ag nanotriangle both had a positive shift of 1.1 and 1.0 eV when mixed with graphene. They explained it as the electron transfer from Ag to graphene [12]. Wu et al. [35] also deduced the Ag 3d peaks displaying nearly 0.6 eV shift to higher binding energy for Ag deposited on graphene sheet is the result of electron transfer from metallic Ag to graphene sheet. With 3 nm etching, a significant positive shift of the binding energy for Ag 3d_5/2_ relative to about 368.4 eV of the bulk Ag° was identified. The above results confirm that the existing state of Ag NPs is related to TiO_2_ and rGO.

**Fig. 2:**
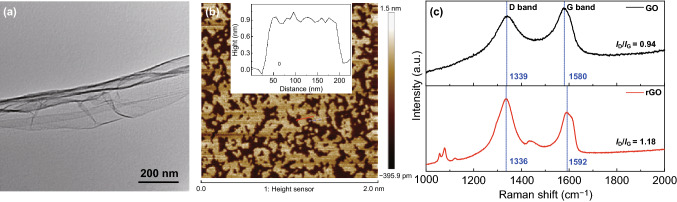
**a** TEM and **b** AFM images of the GO flakes with the thickness distribution shown in the inset. **c** Raman spectra of GO and rGO

TEM and AFM techniques were used to test the structure and size of GO. The TEM image of GO is shown in Fig. 2a, and the ultrathin wrinkles confirm the existence of GO sheets. Through analyzing the GO flakes with AFM (Fig. 2b), the slice sizes were measured in the range of 0–200 nm and the thickness of the thin layer was about 0.9 nm. The reduction of GO in the composites was verified by Raman spectra, as displayed in Fig. 2c, exhibiting the characteristic D and G bands of the carbon materials. The intensity ratio of D to G band (*I*_D_/*I*_G_) can be used to evaluate rGO graphitization degree because it can be increased by the chemical reduction of GO [36]. The result shows that the *I*_D_/*I*_G_ ratio increases from 0.94 of GO to 1.18 of rGO, indicating the successful reduction of GO under UV irradiation [25, 37].

**Fig. 3:**
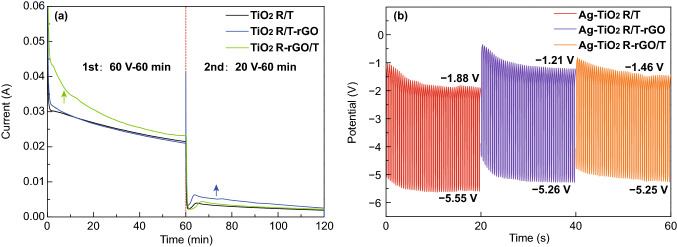
**a** The *I-t* curves in the two-step anodic oxidation process and **b** *U-t* curves in pulse electrodeposition of TiO_2_ hierarchical structure

The as-prepared GO dispersion was dividedly put into the electrolyte of the first and second step anodic oxidation to prepare TiO_2_ R-rGO/T and TiO_2_ R/T-rGO, causing a few differences in *I*-*t* and *U*-*t* of the preparation process. The variation of current versus time is recorded in Fig. 3a; the measured current significantly gets large with GO in electrolyte, such as the current of TiO_2_ R-rGO/T in the first step (green line) and TiO_2_ R/T-rGO in the second step (blue line). The abundant negatively charged functional groups of GO make it moved toward the anode under the action of external potential, facilitating the formation of the TiO_2_ hierarchical structures. On this basis, Ag NPs were deposited on TiO_2_ by pulsed-electrodeposition method. The pulse currents were set as − 0.25 mA of the nucleation processes and 0 mA of the growth processes, and the corresponding *U*-*t* variations are shown in Fig. 3b. In contrast, the potential in the whole process corresponding to Ag-TiO_2_ R/T is the smallest (− 5.55 V, − 1.88 V). It can be concluded that the resistance of the TiO_2_ R/T sample in nucleation stage is the largest and its internal electric field at growth stage is the strongest, which is conducive to grow rather than nucleate of Ag NPs. In other words, the decoration of rGO in TiO_2_ nanorings and nanotubes has reduced the internal resistance of the composites.

**Fig. 4:**
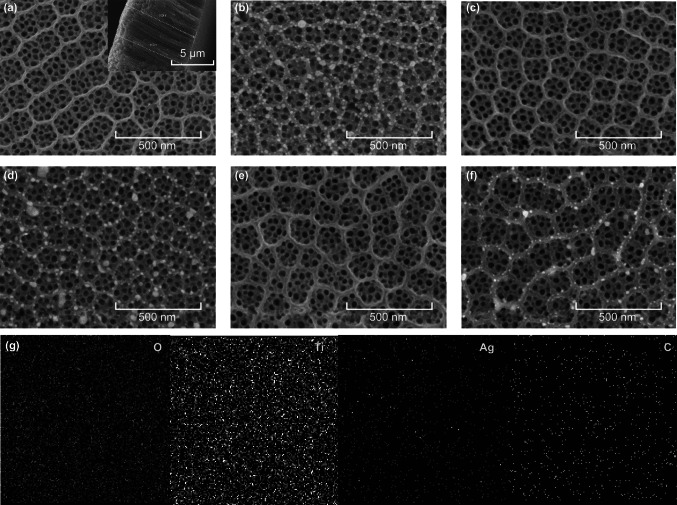
Top view SEM images of TiO_2_-based nanomaterials of **a** TiO_2_ R/T, **b** Ag-TiO_2_ R/T, **c** TiO_2_ R/T-rGO, **d** Ag-TiO_2_ R/T-rGO, **e** TiO_2_ R-rGO/T, **f** Ag-TiO_2_ R-rGO/T. The inset in **a** shows the TiO_2_ R/T cross-sectional views. **g** The mapping images of the Ag-TiO_2_ R/T-rGO

SEM images can clearly reveal the morphologies of TiO_2_ R/T, Ag-TiO_2_ R/T, TiO_2_ R/T-rGO, Ag-TiO_2_ R/T-rGO, TiO_2_ R-rGO/T, and Ag-TiO_2_ R-rGO/T. From Fig. 4a, it can be seen that the hierarchical top-ring/bottom-tube structures were successful fabricated. The average diameter of the nanorings is about 150 nm with 35 nm thickness, and the diameter of nanotubes is about 50 nm. The concaves in the top layer are expected to work as nanomirrors for light reflection and scattering. The insert gives a cross-sectional view, showing that the nanotubes with a length of about 9 μm are perpendicular to the Ti substrate. Figure 4c, e shows the TiO_2_ R/T decorated with rGO in nanotubes and nanorings, respectively. It is seen that the addition of rGO has no apparent effect on the morphology of TiO_2_ R/T, but the electrostatic force between rGO and Ti^4+^ made the nanotubes fairly grown up in the first anodization of TiO_2_ R-rGO/T, thereby taking a long time to remove the film of nanotubes by ultrasound, which is consistent with Fig. 3a [38]. Due to the slow penetration of precursor solution into the air-filled nanotubes, plasmonic Ag NPs were mainly loaded on the nanorings of TiO_2_ R/T [39]. In Fig. 4b, d, the average size of Ag NPs adhered on the top edge of the nanorings is about 20 nm, possessing an optimal quality factor of SPR [40]. Figure 4f shows that the average diameter of Ag NPs deposited on TiO_2_ R-rGO/T has decreased to 6 nm, due to the strong Ag-C bond between Ag and rGO existing in the surface [29]. The electrostatic force is conducive to the uniform formation of Ag NPs on the surface, i.e., promotes the nucleation process. The EDS mapping over Ag-TiO_2_ R-rGO/T was further investigated and presented in Fig. 4g, indicating that all the elements (O, Ti, Ag, and C) were uniformly dispersed.

### The Effect of rGO on the Optical Properties of TiO_2_-Based Composites

**Fig. 5:**
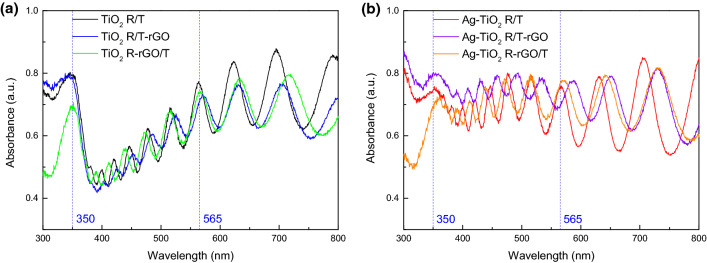
UV-Vis absorption spectra of **a** TiO_2_ R/T, TiO_2_ R/T-rGO, TiO_2_ R-rGO/T and **b** Ag-TiO_2_ R/T, Ag-TiO_2_ R/T-rGO, Ag-TiO_2_ R-rGO/T

Figure 5 shows the UV–Vis absorption spectra of the as-prepared samples. Obviously, all samples show oscillating absorption peaks in the visible region, which are derived from the interference of lights reflecting from the top nanorings and the bottom Ti substrate [30]. It is obvious that the light absorption of TiO_2_ R/T, TiO_2_ R/T-rGO, TiO_2_ R-rGO/T in the range of 350–565 nm is weak, as shown in Fig. 5a. From Fig. 5b, the deposition of Ag has greatly improved the photoabsorption of TiO_2_ in the region, in agreement with the absorption enhancement of 400–650 nm in Ref. [31], which is related to the SPR effect of spatially confined electrons in Ag NPs. It has been reported that the amplitude of oscillating peaks of pristine TiO_2_ R/T increases significantly with similar frequency after annealing, attributed to the formation of defects in TiO_2_ [30]. Here, the absorption peaks of rGO-decorated samples show a slight red shift and the amplitude of oscillation peaks has decreased. Thus, we can infer that the addition of rGO into TiO_2_ nanorings or nanotubes has changed the spatial shapes or interactions of the molecules by introducing defects into TiO_2_, influencing the transition of the valence electrons. Finally, the UV–Vis absorption spectra of TiO_2_ R/T with or without rGO show different performances. In addition, compared with bare TiO_2_ R/T, TiO_2_ R/T-rGO has no significant change, while TiO_2_ R-rGO/T has weakened absorption in 300–350 nm. The same thing happens to Ag-TiO_2_ R/T, Ag-TiO_2_ R/T-rGO, Ag-TiO_2_ R-rGO/T. It is suggested that adding rGO into nanotubes in the second step is a great way to improve the light absorption of TiO_2_ R/T. Taking all samples into account, Ag-TiO_2_ R/T-rGO has the greatest UV–Vis absorption property.

**Fig. 6:**
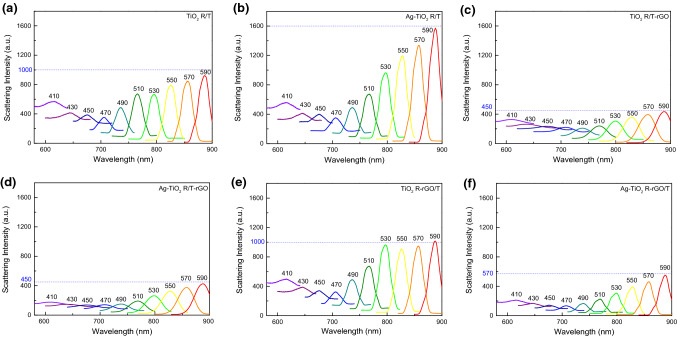
Scattering spectra with different excited wavelength of **a** TiO_2_ R/T, **b** Ag-TiO_2_ R/T, **c** TiO_2_ R/T-rGO, **d** Ag-TiO_2_ R/T-rGO, **e** TiO_2_ R-rGO/T, and **f** Ag-TiO_2_ R-rGO/T

For the spherical metal nanoparticles, the absorption increases with the decrease in scattering [41]. At present, the main controversy regarding the mechanism of the emission is the assignment to either radiative recombination of hot carriers (photoluminescence) or light scattering [42, 43]. Here, we measured the scattering spectra of the TiO_2_-based samples. A broad excitation wavelength range (410–590 nm) was chosen to contain the extinction spectrum of Ag and rGO as far as possible [44]. The final scattering spectra were collected and illustrated in Fig. 6. With the incident wavelength increasing, the scattering intensities of all samples decreased first and then increased, and the maximum scattering intensity corresponded to 900 nm irradiation within the scope of the measurement. In comparison with TiO_2_ R/T, the scattering intensity of Ag-TiO_2_ R/T is enhanced, as shown in Fig. 6a, b. It is known that Ag NPs are helpful to the radiative scattering process induced by coupling to a propagating plasmon mode, so the fluorescence enhancement is observed [45]. For the utilization efficiency of plasmon-excited hot carriers, one of the major limitations is the loss in carrier energy due to carrier scattering within the plasmonic metals [46]. When adding rGO into the electrodes, the drastically weakened scattering of TiO_2_ R/T-rGO and Ag-TiO_2_ R/T-rGO is observed in Fig. 6c, d. On the other hand, compared with TiO_2_ R/T, the scattering intensity of TiO_2_ R-rGO/T corresponding to incident light *λ* > 530 nm is higher (Fig. 6e), but the scattering intensity of Ag-TiO_2_ R-rGO/T has decreased to 570 (Fig. 6f), indicated by the blue dashed line. Anyway, Ag-TiO_2_ R/T-rGO has the weakest scattering intensity, corresponding to the great light absorption as shown in Fig. 5.

**Fig. 7:**
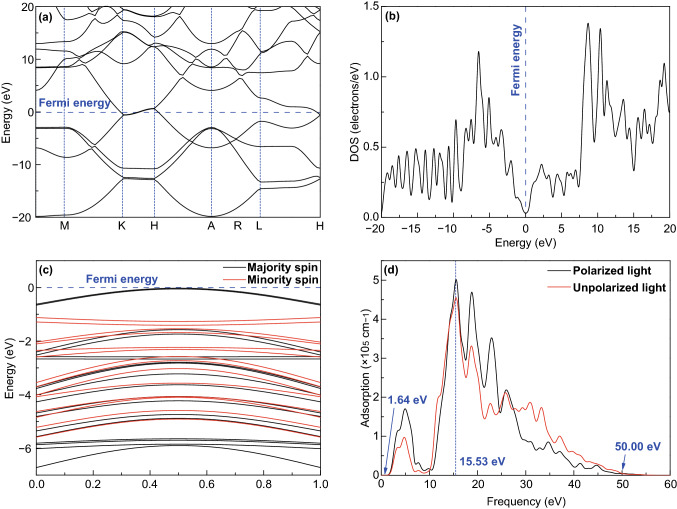
**a** Kohn–Sham band structure, **b** density of states, and **c** band structure of the majority/minority spin of graphene. The Fermi energy is set as zero and indicated by blue dashed line. **d** The absorption spectra under polarized light in the direction of (1, 0, 0) and unpolarized light

To further study the effect of rGO on the optical properties of TiO_2_-based composites, the investigations of rGO using the plane wave ultrasoft pseudopotential method based on the density functional theory were conducted in detail. The Fermi energy is set to zero, represented by the blue dashed line. The computed band structure of rGO (Fig. 7a) matches well with the previous results in Ref. [47], indicating that the character of density of states (DOS) and optical properties are reasonable and reliable. Figure 7b shows the DOS of graphene, suggesting that the electron density in Fermi energy level is the lowest. The band structures of the majority spin and the minority spin were obtained by spin calculation (Fig. 7c). The remarkable difference is that the Fermi level intersects with the majority spin while lying in the band gap of the minority spin. Therefore, graphene has apparent spin polarization and should be regarded as “half metal.” In Fig. 7d, the absorption coefficients of the anisotropic material under polarized and unpolarized light irradiation have a little difference. The absorption range of graphene is 1.64–50.00 eV, corresponding to 756.10–24.80 nm, which is calculated by Eq. 1,

1 $$E = \frac{hc}{\lambda }$$

where *E* is the energy of light (eV), *h* is Planck constant (4.13566743 × 10^−15^ eV s), *c* is the speed of light (3.153 × 10^17^ nm s^−1^), and *λ* is the wavelength of light (nm). Accordingly, rGO can effectively enhance the light absorption of TiO_2_.

### Photoelectrochemical Properties and Hydrogen Production Rates

**Fig. 8:**
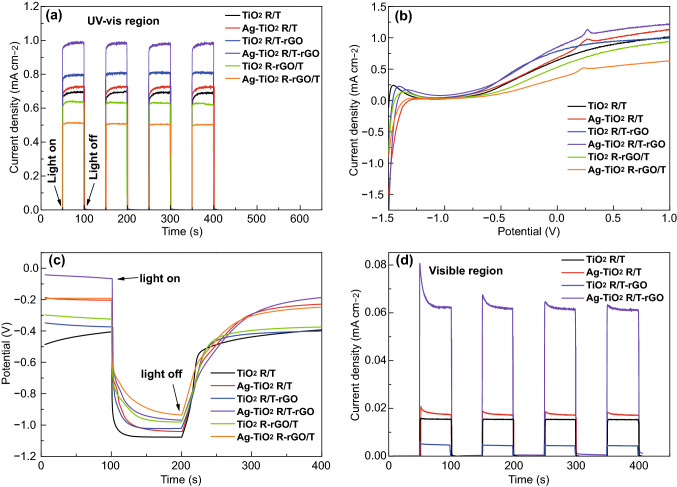
**a** Photocurrent responses in the light on–off process under illumination of AM 1.5, **b** Linear sweep voltammetry, **c** open-circuit photovoltage curves, and **d** photocurrent responses in the light on–off process under illumination of visible light (*λ* > 400 nm) of TiO_2_-based nanomaterials

Chronoamperometric *I*-*t* curves are recorded in Fig. 8a by irradiating the electrodes with AM 1.5, which are measured to investigate the transient photocurrent response. Upon illumination, the photocurrents of all photoanodes can rapidly reach a steady state and revert to zero when light turns off. All samples exhibit fast photocurrent response and stable photocurrent density under chopped light. The maximum photocurrent density is 0.98 mA cm^−2^ of Ag-TiO_2_ R/T-rGO, which is almost 1.5 times of bare TiO_2_ R/T (0.69 mA cm^−2^). The photocurrents of TiO_2_ R/T-rGO (0.80 mA cm^−2^) and Ag-TiO_2_ R/T (0.73 mA cm^−2^) are also greater than TiO_2_ R/T, but TiO_2_ R-rGO/T (0.63 mA cm^−2^) and Ag-TiO_2_ R-rGO/T (0.51 mA cm^−2^) have smaller photocurrent density.

To characterize the photocurrent density as a function of applied voltage, a set of LSV curves are presented in Fig. 8b, measured with the applied potential window of -1.5–1.0 V. The obtained photocurrent densities at 0 V of the considered six samples lie in the following order: Ag-TiO_2_ R/T-rGO > TiO_2_ R/T-rGO > Ag-TiO_2_ R/T > TiO_2_ R/T > TiO_2_ R-rGO/T > Ag-TiO_2_ R-rGO/T. It is consistent with the measured photocurrent densities under AM 1.5 shown in Fig. 8a. In addition, the apparent oxidation peaks of Ag-TiO_2_ R/T, Ag-TiO_2_ R/T-rGO, and Ag-TiO_2_ R-rGO/T at about 0.25 V correspond to the transformation of Ag^0^ into Ag^+^. Figure 8c shows the open-circuit photovoltage curves, suggesting the n-type conductivity of all samples [48]. The negative increase in voltage under light irradiation suggested that the photogenerated electrons are injected from the semiconductor film into the Ti substrate [49]. The differences of the potential in light and dark are calculated as 0.68, 0.84, 0.65, 0.90, 0.65, and 0.74 V, corresponding to TiO_2_ R/T, Ag-TiO_2_ R/T, TiO_2_ R/T-rGO, Ag-TiO_2_ R/T-rGO, TiO_2_ R-rGO/T, and Ag-TiO_2_ R-rGO/T, respectively. The open-circuit potential has increased with the loading of Ag NPs, while a slight decrease of about 0.03 V has been observed with the addition of rGO in nanotubes and nanorings. All above results show that the strategy of designing Ag-TiO_2_ R/T-rGO nanostructure is efficient for promoting the photoelectric properties of the PEC system. Combined with the images shown in Fig. 4d, f, the Ag NPs deposited on TiO_2_ R/T-rGO and TiO_2_ R-rGO/T are 20 and 6 nm, respectively. Therefore, although rGO can reduce the size of Ag NPs, the TiO_2_ composite electrode deposited by Ag NPs with the strongest SPR effect has better photoelectric performance.

In accordance with the UV–Vis absorption spectra (Fig. 5), the enormous enhancement of photocurrent density under visible light (*λ* > 400 nm) illumination is observed in Fig. 8d. Obviously, Ag-loaded samples give quick response and then get down to the stable values. The interconversion of Ag/Ag^+^ during the alternating irradiation ensures a stable visible light-induced photocurrent [50]. The maximum photocurrent density is 0.06 mA cm^−2^ of Ag-TiO_2_ R/T-rGO, followed by 0.017 mA cm^−2^ of Ag-TiO_2_ R/T, 0.015 mA cm^−2^ of TiO_2_ R/T, and 0.005 mA cm^−2^ of TiO_2_ R/T-rGO. Thus, Ag-TiO_2_ R/T-rGO hybrid exhibits a fourfold increased current density under visible light compared with pristine TiO_2_ R/T.

**Fig. 9:**
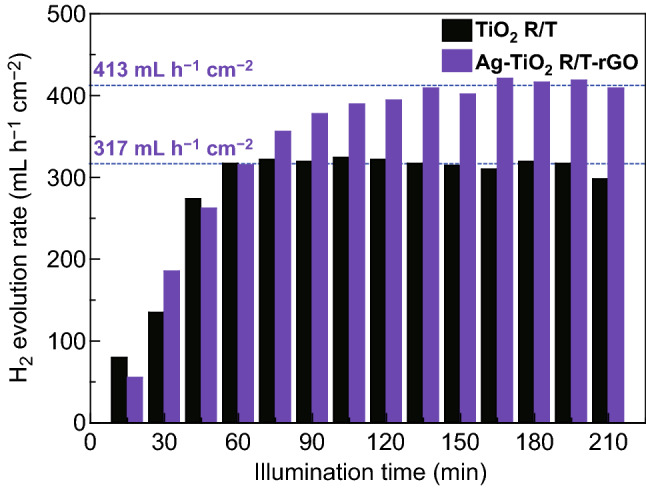
Photocatalytic H_2_ production. The hydrogen evolution rate was measured by using the TiO_2_ R/T and Ag-TiO_2_ R/T-rGO samples as photoanodes and Pt foil as cathode in a PEC cell containing a 0.5 M Na_2_SO_4_ solution under AM 1.5

At last, we tested the hydrogen generation performance of Ag NPs and rGO enhanced hierarchical TiO_2_ nanoring/nanotube arrays via choosing pristine TiO_2_ R/T for comparison. The electrolyte was deaerated with N_2_ for 3–4 h until the dissolved oxygen could be neglected. Once the tested three-electrode system exposed on simulated sunlight, the rate of H_2_ evolution rapidly increased during the first hours and remained nearly constant afterward, as described in Ref. [51]. The responding time of TiO_2_ R/T and Ag-TiO_2_ R/T-rGO is 60 and 120 min, respectively. As displayed in Fig. 9, Ag-TiO_2_ R/T-rGO electrode exhibits excellent photoelectrochemical water splitting activity under AM 1.5, with an enhanced average H_2_ evolution rate of 413 μL h^−1^ cm^−2^. This value exceeds by 1.30 times than that of TiO_2_ R/T (317 μL h^−1^ cm^−2^). It remains stable during the test, partly due to the suitable conduction band edge of rGO, which is high enough to make H_2_ generated [52, 53].

### The Charge Transfer and Proposed Mechanisms of TiO_2_-Based Electrodes with Decoration of Ag and rGO

Intensity-modulated photocurrent spectroscopy (IMPS) is usually used to examine the recombination of conduction band electrons at surface states and charge transfer to solution. Here, we used IMPS to clarify the relationship of the charge transfer with SPR effect of Ag as well as rGO. From the IMPS complex plane plots, the electron-transport time ($\tau_{\text{d}}$) can be calculated with the frequency ($f_{ \hbox{min} }$) at the zenith of the semicircle through Eq. 2,

2 $$\tau_{d} = \frac{1}{{2\pi f_{\hbox{min} } }}$$

**Fig. 10:**
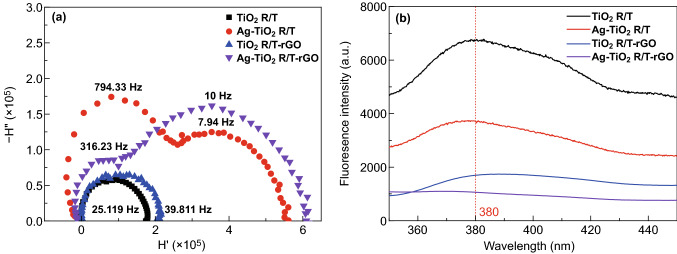
**a** IMPS plots and **b** PL spectra with the excited wavelength at 260 nm of TiO_2_ R/T, Ag-TiO_2_ R/T, TiO_2_ R/T-rGO, and Ag-TiO_2_ R/T-rGO

As shown in Fig. 10a, there is one distinct semicircle for TiO_2_ R/T and TiO_2_ R/T-rGO, and their electron-transport time is calculated as 6.34 and 4.00 ms, respectively. Ag-TiO_2_ R/T and Ag-TiO_2_ R/T-rGO exhibit two semicircles, implying two different electron-transport modes [54]. As for Ag-TiO_2_ R/T, a fraction of the electrons were collected with a short transport time (0.20 ms), while the remaining electrons were collected with a long time (20 ms). The electron-transport time of Ag-TiO_2_ R/T-rGO also contains two parts, and the total time equals to 16.42 ms, which is shorter than that of Ag-TiO_2_ R/T. Thus, rGO can accelerate the charge transfer over TiO_2_ nanotube arrays. It is helpful for the migration of plasmon-induced hot electrons from Ag to TiO_2_.

By analyzing the photoluminescence (PL) spectra of the TiO_2_-based composites, the lifetime of the photogenerated electron–hole pairs in semiconductors under irradiation can be obtained. The greater the photoluminescence emission, the more recombination of electron–hole pairs. Thus, the Ag-TiO_2_ R/T-rGO sample exhibits lower electron–hole recombination rate, as revealed by its weakened PL peak (Fig. 10b). At the same time, Ag-TiO_2_ R/T and TiO_2_ R/T-rGO also show a lower recombination rate than TiO_2_ R/T, owing to the trapping of the photo-excited electrons of Ag NPs and the high charge carrier mobility of rGO, respectively [17].

**Fig. 11:**
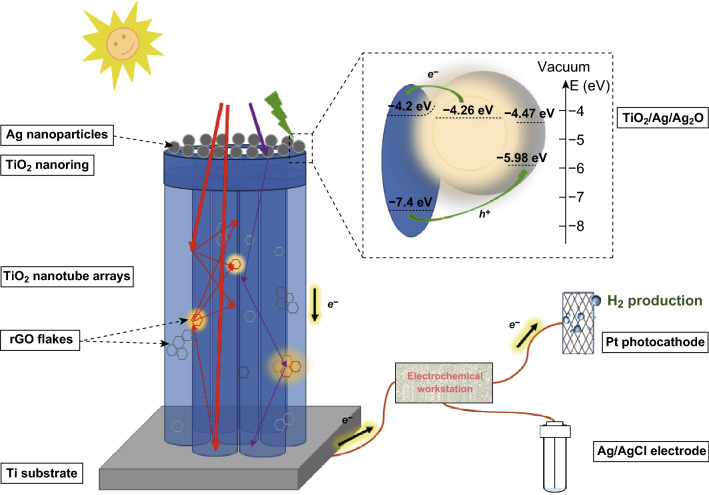
Schematic illustration of the photoresponse and photogenerated charge transfer of Ag-TiO_2_ R/T-rGO under UV–Vis light irradiation

On the basis of the discussion above, the photoresponse and charge transfer play an important role in PEC hydrogen production via water splitting. Therefore, the proposed mechanisms of Ag-TiO_2_ R/T-rGO are schematically illustrated in Fig. 11. In this picture, the gray sphere and hexagon represent Ag NPs and rGO flakes, respectively. On the one hand, UV light (purple arrows), which can be absorbed by TiO_2_, and visible light (red arrows) interfere with each other in the nanotubes producing oscillation peaks, as shown in Fig. 5. Part of the photogenerated electrons is transferred to Ag NPs to reduce the loss of holes in recombination process. Meanwhile, the light irradiated on rGO is absorbed (turn yellow). On the other hand, the light in the range of 350–565 nm (green arrows) is absorbed by Ag NPs (turn yellow) through the SPR excitation, and then, the hot electrons are injected into the conduction band of TiO_2_ (*E*_cd_ = − 4.2 eV) over the Schottky barrier [5, 9]. The wall of TiO_2_ nanotubes possesses excellent ability to transfer electrons, allowing more electrons to be trapped by H^+^ with the formation of H_2_ [36]. Moreover, the surface of the Ag NPs has inevitably oxidized to Ag_2_O (*E*_cd_ = − 4.47 eV, *E*_vd_ = − 5.98 eV), according to Fig. 1c. Because the energy of the electrons in the conduction band is insufficient to reduce a proton, Ag_2_O serves as an electrocatalyst for the oxidation reaction of photoelectrical water splitting [55, 56]. Eventually, both of the photogenerated electrons and hot electrons are transported through Ti substrate to Pt photocathode to perform the hydrogen evolution reaction, and yet holes are enriched synchronously on Ag_2_O to oxidize water into O_2_.

## Conclusions

In summary, we designed the hybrid Ag-TiO_2_-rGO catalyst structure to realize enhanced wide spectrum photocatalytic hydrogen evolution and mainly researched the great influence of rGO on Ag and TiO_2_. For pristine TiO_2_ R/T and TiO_2_ R/T with rGO in nanotubes, 20 nm Ag NPs were uniformly deposited on the surface. When adding rGO into TiO_2_ nanorings, the size of the deposited Ag NPs has reduced to 6 nm due to the Ag-C bond between Ag and rGO. With the ternary composites formed, the electrons transfer from Ag to rGO was identified by the positively shifted of the Ag 3d peaks in XPS spectra. The deposition of Ag NPs on TiO_2_ R/T can enhance the absorption in 350–565 nm region, but the light scattering of Ag-decorated samples was also enhanced attributed to the propagating plasmon mode. Fortunately, the introduction of rGO can greatly weaken the scattering due to its broad absorption in UV–Vis light region, verified by the density functional theory calculations. On the other hand, IMPS illustrates that rGO can promote the transportation of hot electrons to Ti substrate, resulting in the reduction of photogenerated electron–hole recombination, as shown in PL plots. By comparison, Ag-TiO_2_ R/T-rGO photoelectrode shows the largest photocurrent density and open-circuit potential under AM 1.5 irradiation. Meanwhile, the remarkable photocurrent under visible light irradiation highlights its excellent performance in visible region. Finally, Ag-TiO_2_ R/T-rGO photoelectrode resulted in a remarkable boost in the H_2_ evolution rate (413 μL h^−1^ cm^−2^) compared to pristine TiO_2_ R/T photoelectrode (317 μL h^−1^ cm^−2^). Collectively, our work opens a new window for the novel design and synthesis of rGO-contained plasmonic photoelectrode, which is conducive to give full play to the excellent performance of rGO.
